# An organizing principle for two-dimensional strongly correlated superconductivity

**DOI:** 10.1038/srep22715

**Published:** 2016-03-11

**Authors:** L. Fratino, P. Sémon, G. Sordi, A.-M. S. Tremblay

**Affiliations:** 1 Department of Physics, Royal Holloway, University of London, Egham, Surrey, UK, TW20 0EX; 2 Département de physique and Regroupement québéquois sur les matériaux de pointe, Université de Sherbrooke, Sherbrooke, Québec, Canada J1K 2R1; 3 Canadian Institute for Advanced Research, Toronto, Ontario, Canada, M5G 1Z8

## Abstract

Superconductivity in the cuprates exhibits many unusual features. We study the two-dimensional Hubbard model with plaquette dynamical mean-field theory to address these unusual features and relate them to other normal-state phenomena, such as the pseudogap. Previous studies with this method found that upon doping the Mott insulator at low temperature a pseudogap phase appears. The low-temperature transition between that phase and the correlated metal at higher doping is first-order. A series of crossovers emerge along the Widom line extension of that first-order transition in the supercritical region. Here we show that the highly asymmetric dome of the dynamical mean-field superconducting transition temperature 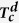, the maximum of the condensation energy as a function of doping, the correlation between maximum 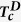 and normal-state scattering rate, the change from potential-energy driven to kinetic-energy driven pairing mechanisms can all be understood as remnants of the normal state first-order transition and its associated crossovers that also act as an organizing principle for the superconducting state.

In hole-doped cuprate high-temperature superconductors, d-wave superconductivity shows unusual features that cannot be explained by theoretical methods based on weak correlations^1^,^2^. This has motivated the hypothesis that such unusual features emerge from doping a two-dimensional Mott insulator. Advances in this regard were enabled by the development of new theoretical methods such as cluster extensions^3^,^4^ of dynamical mean-field theory^5^. A collective effort over the last decade has shown that the key aspects of the phenomenology of cuprates are contained in the two-dimensional Hubbard model. Within this theoretical framework, here we show that these key aspects rest with a single organizing principle, namely a normal-state first-order transition between pseudogap and correlated metal beneath the superconducting dome, identified in ref. 6. Our analysis indicates that this emerging phase transition at finite doping shapes not only the normal-state phase diagram, but strikingly leaves its mark on the complex structure of the superconducting condensate that is born out of this unusual normal state.

## Model and Method

The two dimensional Hubbard model on a square lattice reads


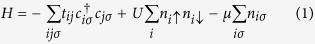


where 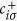 and *c*_*iσ*_ operators create and destroy an electron of spin *σ* on site *i*, *n*_*iσ*_ = 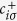 *c*_*iσ*_ is the number operator, *μ* is the chemical potential, *U* the onsite Coulomb repulsion and *t*_*ij*_ is the nearest neighbor hopping amplitude. Neglecting second-neighbor hopping, necessary to capture the correct Fermi surface, minimizes the Monte-Carlo sign-problem and does not alter our main findings (see supplementary Fig. S7). Unless specified, the lattice spacing, Planck’s constant, Boltzmann’s constant and *t* are unity.

We solve this model using cellular dynamical mean-field theory^3^,^4^ (CDMFT) on a 2 × 2 plaquette immersed in an infinite self-consistent bath of non-interacting electrons. This plaquette is the minimal cluster that includes all two-dimensional short-range charge, spin and superconducting dynamical correlations. We do not take into account long-range charge-density waves in light of the recent experimental results where this transition is removed by pressure^7^. Long-range antiferromagnetism concomitant with long-range superconductivity has been treated at *T* = 0 in previous work^8^,^9^,^10^. Since we are interested in large values of *U*, i.e. a doped Mott insulator, the most appropriate method to solve the impurity (cluster plus bath) problem is the hybridization expansion continuous-time quantum Monte Carlo method^11^. Sign problems prevent the study of large *U* with alternate quantum Monte Carlo methods^11^. We use two recent algorithmic improvements to speed up the calculations: a fast rejection algorithm with skip-list data structure^12^ and four point updates that are necessary for broken symmetry states like d-wave superconductivity^13^.

Let us first consider the superconducting phase diagram. We then discuss features of the normal state that determine its shape.

### Superconducting dome

**Figure 1:**
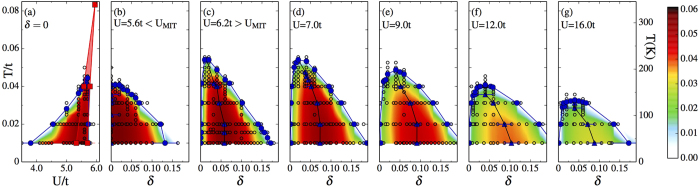
d-wave superconducting phase obtained by the plaquette CDMFT solution of the two-dimensional Hubbard model. We explore the *T* − *U* − *δ* space by taking cuts at *n* = 1 as a function of *U* and *T* [panel (**a**)] and at constant *U* as a function of *δ* and *T* [panels (**b–g**)]. Superconductivity is delimited by 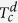 (line with blue filled circles), the temperature below which the superconducting order parameter Φ is nonzero. Color corresponds to the magnitude of |Φ| (see supplementary Fig. S1 for Φ(*U*) and Φ(*δ*) curves at different *T*). The loci of Φ_max_(*δ*) are shown by blue triangles. On the right vertical axis we convert temperature to Kelvin by using *t* = 0.35eV. The coexistence region across the first-order Mott metal-insulator transition appears in panel (**a**) as red shaded area. It is obtained from the hysteretic evolution of the double occupancy with U^14^.

Previous studies show that both at half-filling and at finite doping the metallic state close to the Mott insulator is unstable to d-wave superconductivity^8^,^9^,^10^,^14^,^15^,^16^,^17^,^18^,^19^,^20^,^21^,^22^,^23^. In Fig. 1 we map out the superconducting state in the *U* − *T* plane for the undoped case and in the *δ* − *T* plane for different values of *U*. The superconducting region is defined as the region of non-zero superconducting order parameter 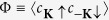 (where the cluster momentum **K** is (*π*, 0)). The boundary, 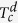, is obtained from the mean of the two temperatures where Φ changes from finite to a small value (here |Φ| = 0.002). While there is no continuous symmetry breaking in two dimensions at finite temperature, 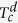 physically denotes the temperature below which the superconducting pairs form within the cluster^14^. The actual *T*_*c*_ can be reduced (because of long wavelength thermal or quantum fluctuations^24^ or of competing long range order^1^) or increased (because of pairing through long wavelength antiferromagnetic fluctuations^25^), but 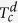 still remains a useful quantity marking the region where Mott physics and short-range correlations produce pairing.

As a function of *U*, 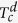 changes from finite to zero discontinuously at the first-order Mott metal-insulator transition (red shaded region in panel a). Superconductivity appears in the metastable metallic state near the Mott insulator, never in the Mott insulator itself (panels a, b). As a function of doping, 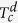 forms a dome as long as *U* is larger than the critical value necessary to obtain a Mott insulator at half-filling (panels c–g). In our previous studies^13^,^14^ we left opened two possibilities: as a function of *δ*, either superconductivity is separated from the Mott insulator at *δ* = 0 by a first-order transition or there is an abrupt fall of 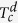
(*δ*). By increasing the resolution in doping near *δ* = 0, here we find the latter, namely *T*_*c*_^*d*^(*δ*) plummets with decreasing *δ*.

The superconducting dome is highly asymmetric. 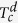 (*δ*) is zero at *δ* = 0, initially rises steeply with increasing *δ*, reaching a peak at the optimal doping *δ*_opt_ and then declines more gently with further doping. The global maximum *T*_*c*_^max^ of 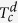 in the *U* − *δ* − *T* space occurs just above *U*_MIT_ and at finite doping *δ*_opt_. Further increase of *U* leads to a decrease in 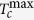, as expected if 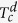 (*δ*) scales with the superexchange energy *J* = 4*t*^2^/*U* for large enough *U*^10^,^26^. As a function of *U*, the optimal doping *δ*_opt_ departs from *δ* = 0 for *U* > *U*_MIT_, increasing with increasing *U* and saturating around *δ* ≈ 0.04 for large *U* (see also supplementary Fig. S2).

The range of doping where superconductivity occurs at the lowest temperature is consistent^13^ with results obtained with CDMFT at *T* = 0^10^. The asymmetric superconducting dome with an abrupt fall of 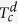 with decreasing *δ* is also consistent with dynamical cluster approximation results on larger clusters^22^. In the latter calculations, the increased accuracy in momentum space leads to a *T*_*c*_ that vanishes before half-filling.

### Superconducting order parameter

To analyse the shape of the superconducting phase we turn to the superconducting order parameter Φ, whose magnitude is color-coded in Fig. 1 (the raw data is in Fig. S1). While 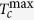 occurs at finite doping, the overall maximum Φ_max_ is found in the undoped model close to the Mott insulator. But as a function of doping, for *U* > *U*_MIT_, Φ forms a dome that reaches a peak at *δ*_Φ max_. At our lowest temperature, *δ*_Φmax_ increases with increasing *U*, and saturates around *δ* ≈ 0.11^10^ for large values of *U*. Notice that *δ*_Φ max_ at our lowest temperature does not coincide with *δ*_opt_, i.e. the doping that optimizes 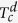. Hence, 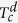 (*δ*) does not scale with Φ(*δ*, *T* → 0). Instead, the locus of the maxima of Φ in the *δ* − *T* plane at fixed *U* traces a negatively sloped line within the superconducting dome (lines with blue triangles) that separates the superconducting dome in two regions. The sharp asymmetry of the superconducting dome is thus linked to this negatively sloped line, which in turn is related to the phase transition between pseudogap and correlated metal in the underlying normal state, as we discuss below.

### Superconductivity and pseudogap

**Figure 2:**
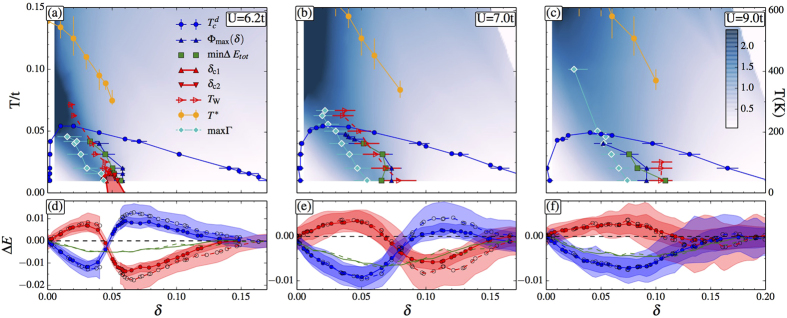
(**a**–**c**) Temperature versus hole doping phase diagram for *U*/*t* = 6.2, 7 and 9, respectively. Superconductivity is delimited by 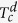 (line with blue filled circles). Beneath the superconducting dome, the normal-state coexistence region across the first-order transition between a pseudogap and a correlated metal appears in (**a**) as red shaded area. It is delimited by the jumps in the electron density as a function of chemical potential and collapses at the critical endpoint (*T*_*p*_, *δ*_*p*_). The Widom line *T*_*W*_ emerging from the endpoint is estimated by the maxima of the charge compressibility along paths at constant *T* (line with red triangles)^6^, and the pseudogap onset T^*^ is computed by the maximum of the spin susceptibility (line with orange circles)^28^. The loci of Φ_max_(*δ*) are shown by blue triangles and follow *T*_*W*_ of the underlying normal state. Color corresponds to the magnitude of the scattering rate Γ, estimated from the zero-frequency extrapolation of the imaginary part of the (*π*, 0) component of the cluster self-energy^29^,^31^. (**d**–**f**) Difference in kinetic, potential and total energies (blue, red and green lines respectively) between the superconducting and normal states, for *T*/*t* = 1/50, 1/100 (full and dashed line, respectively). Shaded bands give standard errors. The loci where the condensation energy is largest are shown in the upper panels as green filled squares. They follow *T*_*W*_(*δ*) and Φ_max_(*δ*).

Understanding the normal state has long been considered a prerequisite to a real understanding of high-temperature superconductivity. This comes out clearly from our results. Previous normal-state CDMFT studies show that for *U* > *U*_MIT_ and small *δ*, large screened Coulomb repulsion *U* and the emergent superexchange *J* lead at low *T* to a state with strong singlet correlations. That phase has the characteristics of the pseudogap phase^6^. The fall of the Knight shift as a function of temperature^27^ is usually associated with T*(*δ*) the onset temperature for the pseudogap. The line with orange filled circles in Fig. 2(a–c) 28 indicates the onset of the drop of the spin susceptibility and of the density of states as a function of *T* and the minimum in the *T* dependence of the *c*-axis resistivity^28^ and is thus T*(*δ*) in our calculation. From our point of view, it is just a precursor to a more fundamental phenomenon. T*(*δ*) exists only if the doping is less than a critical value *δ* < *δ*_*p*_ which is the doping for the critical endpoint (*δ*_*p*_, *T*_*p*_) of a first-order transition that appears in Fig. 2a. A number of crossover lines are associated with this first-order transition. We will discuss them in turn. For larger values of *U*, Fig. 2b,c, the first-order transition is no-longer visible at accessible temperatures, but the crossovers that are left suggest that it is still present^29^.

The normal-state first-order transition separating a pseudogap phase and a correlated metal persists up to the critical endpoint, beyond which only a single normal-state phase exists. Quite generally, different response functions have maxima defining crossover lines emerging from the critical endpoint^30^. The Widom line is known as the line where these maxima join asymptotically close to the critical endpoint^30^. Here we estimate that line, (red open triangles) *T*_W_ in the upper panels of Fig. 2, as the line where the isothermal electronic compressibility has a maximum^6^,^29^,^31^. Let us briefly consider the other crossover lines. A scan in doping at fixed *T* shows that the local density of states at the Fermi energy, the spin susceptibility and the c-axis DC conductivity go through an inflection point at *T*_W_(*δ*)^28^. The first-order transition is also a source of anomalous scattering^29^,^31^. The blue open diamonds indicate the maximum Γ_max_ of the normal state scattering rate Γ. Its magnitude, estimated from the zero-frequency extrapolation of the imaginary part of the (*π*, 0) component of the cluster self-energy, is color-coded in Fig. 2a–c. The region where Γ is large is dark blue. It originates at the transition, extends well above 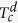 and is tilted towards the Mott insulator. This large Γ is suppressed upon entering the superconducting state^21^,^32^ (see supplementary Fig. S3).

Even though the first-order transition is absent in the superconducting state, the structure it imposes on the normal state shapes the superconducting phase diagram: (a) the maximum of the superconducting order parameter Φ_max_ (line with blue filled triangles in Fig. 2a–c) parallels *T*_*W*_ and Γ_max_, hence the highly asymmetric shape of the superconducting dome is correlated with the slope of the first-order transition and of its supercritical crossovers in the *T* − *δ* plane; (b) Γ_max_ crosses the superconducting dome approximately at *δ*_opt_, hence a region of anomalous scattering broadens as it comes out of the dome; (c) since T* can be detected for doping smaller than *δ*_*p*_ only, superconductivity and pseudogap are intertwined phenomena: superconductivity can emerge from a pseudogap phase below *δ*_*p*_, or from a correlated metal above *δ*_*p*_^14^; (d) the normal state also controls the source of condensation energy, as we now discuss.

### Condensation energy

The superconducting state clearly has a lower free energy than the normal state out of which it is born. In the ground state, the energy difference between both states is known as the condensation energy. The origin of the condensation energy is unambiguous only within a given model^33^,^34^. In the BCS model, superconductivity occurs because of a decrease in potential energy. The kinetic energy increase due to particle-hole mixing in the ground state is not large enough to overcome the potential energy drop. In the cuprates, analysis of inelastic neutron scattering^35^ has suggested that superconductivity arises because of a gain in exchange energy in the *t* – *J* model. Analysis of ARPES^36^ and optical data^37^,^38^,^39^,^40^ in the context of the Hubbard model has suggested that superconductivity is kinetic-energy driven in the underdoped regime^34^,^35^,^41^,^42^,^43^.

In the lower panels of Fig. 2 we plot, for the Hubbard model Eq. 1, the difference in kinetic and potential energies between the superconducting and normal states (Δ*E*_kin_ and Δ*E*_pot_; blue and red lines respectively) as a function of doping. The results for the two different temperatures are close enough to suggest we are close to ground state values. The net condensation energy, shown by the green line, is always negative, as expected. The doping dependence of Δ*E*_kin_ and Δ*E*_pot_ on the other hand shows two striking features: it is non monotonic and can display a sign change. For *U* = 6.2,7, Fig. 2d,e, superconductivity is kinetic-energy driven at small doping and potential energy driven, as in BCS theory, at large doping. For *U* = 9, Fig. 2f, superconductivity is kinetic energy driven for all dopings, although the potential energy difference Δ*E*_pot_ can change sign.

Previous investigations^23^,^39^,^44^ have revealed a complex behavior that remained to this day a puzzle, with Δ*E*_kin_ going from negative to positive depending on *T* and *U*. What has beeno missing to make sense of this complexity is the existence of the normal state first-order transition and its associated supercritical crossovers. By considering different values of *U*, we provide a unified picture of a host of apparently contradictory results. For all *U* considered, the largest condensation energy (see green line in bottom panels of Fig. 2 and green squares in top panels of Fig. 2) is concomitant with the largest superconducting order parameter Φ(*δ*) (but not with the maximum 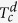) and hence correlates with the normal-state pseudogap-to-correlated metal first-order transition, and its associated supercritical crossovers. For all *U*, the sign changes are also close to the maximum condensation energy and hence also correlated with the same normal-state features. The influence of Mott and superexchange physics extends unambiguously all the way to the normal-state first-order transition terminating at the critical endpoint, from which supercritical crossovers emerge^31^. This reflects itself in the superconducting state in a decisive manner: the changes in sign of the different sources of condensation energy occur for dopings similar to those where the normal-state transition occurs.

### Source of condensation energy

Bottom panels of Fig. 2 (see also Fig. S5) show that in the underdoped region, the kinetic-energy change in the superconducting state is close to minus twice the potential energy change. This is what is expected if superexchange^45^ *J* drives superconductivity there^26^. The decrease with *U* of the maximum 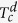, of the magnitude of the individual kinetic and potential energy contributions to condensation energy, and of the maximum value of the *T* = 0 order parameter^8^,^9^,^10^,^18^, are also all consistent with the importance of *J* in the effective model that arises from the Hubbard model at large *U*. The BCS-like behavior in the overdoped regime for *U* = 6.2, 7 probably arises from leftover of the weak-coupling long-wavelength antiferromagnetic spin-wave pairing mechanism^46^, although the effect of the self-consistent rearrangement of the spin-fluctuation spectrum in the superconducting state has not been studied yet.

## Discussion

Our findings further broaden our understanding of the CDMFT solution of the Hubbard model in the doped Mott insulator regime by showing how and to what extent the organizing principle for both the normal state and the superconducting state is the finite-doping first-order transition that determines the shape and the properties of both phases, even though the transition itself is invisible in the superconducting state. In the *T* − *δ* plane, the loci of the maximum order parameter, of the extremum condensation energy, of the maximum normal state scattering relative to the maximum 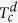, all correlate with crossover lines of the underlying normal state that is unstable to d-wave superconductivity.

We speculate that the application of a magnetic field strong enough to suppress *T*_*c*_ and pressures large enough to remove density waves may reveal the underlying transition. We also speculate that sound anomalies associated with the large compressibility in the underlying normal state above the critical endpoint could appear, in analogy with what is observed near the half-filled Mott transition in layered organics^47^,^48^,^49^,^50^,^51^,^52^. The appearance of large electronic compressibility near the normal state first-order transition suggests that further studies of ubiquitous bond-density waves^7^ should be undertaken with the same set of methods.

## Additional Information

**How to cite this article**: Fratino, L. *et al.* An organizing principle for two-dimensional strongly correlated superconductivity. *Sci. Rep.* **6**, 22715; doi: 10.1038/srep22715 (2016).

## Supplementary Material

Supplementary Information
