# Supplementary material for: An organizing principle for two-dimensional strongly correlated superconductivity
Source: Sci Rep. 2016 Mar 11;6:22715. doi: 10.1038/srep22715 (PMC4786811; doi:10.1038/srep22715)
Supplement: Supplementary Information [file srep22715-s1.pdf]

Supplementary information: An organizing principle for two-dimensional strongly correlated superconductivity

L. Fratino,[1] P. Sémon,[2] G. Sordi,[1] and A.-M. S. Tremblay[2,3]

[1] *Department of Physics, Royal Holloway, University of London, Egham, Surrey, UK, TW20 0EX*
[2] *Département de physique and Regroupement québéquois sur les matériaux de pointe, Université de Sherbrooke, Sherbrooke, Québec, Canada J1K 2R1*
[3] *Canadian Institute for Advanced Research, Toronto, Ontario, Canada, M5G 1Z8*

In this supplementary information, we first remove ambiguities that might arise from color coding in Fig. 1 and Fig. 2 of the main text by plotting the corresponding raw data, first for the superconducting order parameter in Sec. A. The location of the maximum T_c, of the maximum order parameter and of the end of the superconducting dome as a function of doping is also given. The scattering rate Γ is in Sec. B. We also show in this section that the scattering rate decreases drastically in the superconducting state, consistent with the reappearance of quasiparticles in that state. Sec. C summarises the main crossover lines in the normal state found in previous work [1, 2]. We show in Sec. D how the contribution to the kinetic energy from the plaquette can be isolated from more long-distance related contributions. The plaquette contribution can be computed purely from the 4 site density matrix. It will be shown that the latter contribution to the condensation energy is always negative, namely the superconducting state always lowers the plaquette kinetic energy. Finally, Sec. E reports the the $T-\delta$ phase diagram for the second neighbor hopping t', to show that main findings of the main text are not altered by t'.

A. SUPERCONDUCTING ORDER PARAMETER

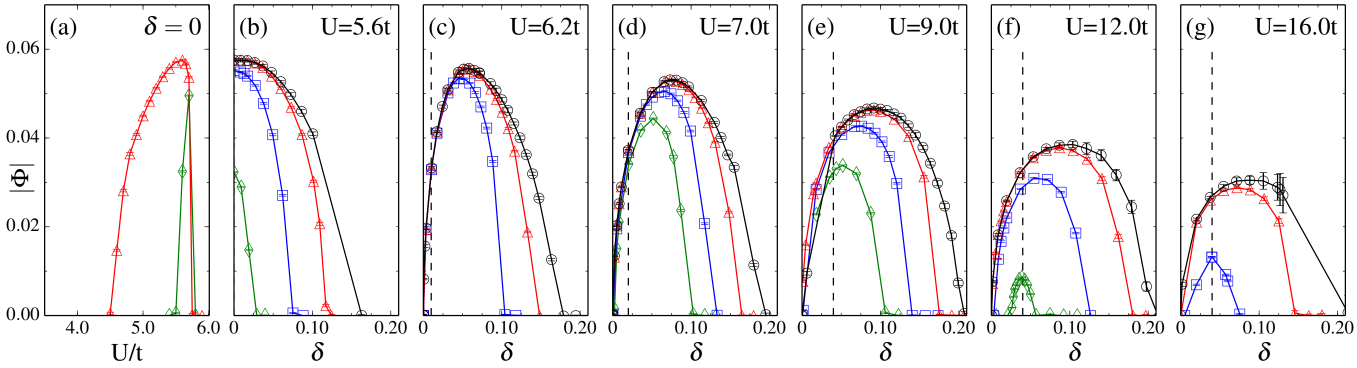

FIG. S1. Superconducting order parameter $|\Phi|$ as a function of U/t [panel (a)] and as a function of δ for several values of the interaction strength U/t [panels (b) to (g)]. The data are shown for temperatures $T/t = 1/25$ (green diamonds), 1/32 (blue squares), 1/50 (red triangles) and 1/100 (black circles). Interpolation of these data gives rise to the color map in Fig. 1 of main text. Dashed vertical line displays the optimal doping $\delta_{\rm opt}$.

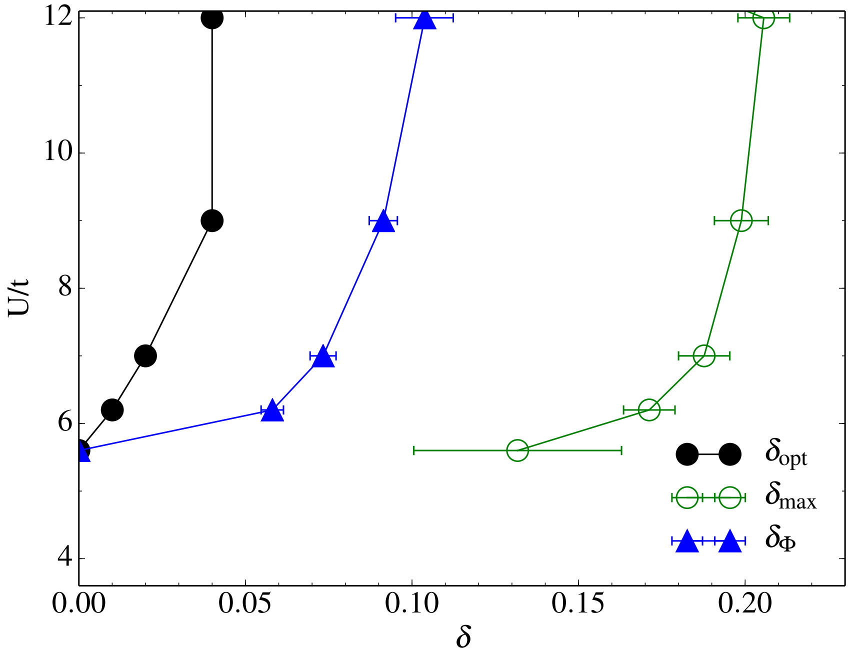

FIG. S2. Characteristic dopings in the $U-T$ plane: optimal doping ($\delta_{\rm opt}$, black circles), the position of the maximum order parameter for $T=1/100$ ($\delta_{\Phi_{\rm max}}$, blue triangles) and the largest doping at which superconductivity disappears for the lowest temperature studied, i.e. $T/t=1/100$ ($\delta_{\rm max}$, green circles).

B. SCATTERING RATE

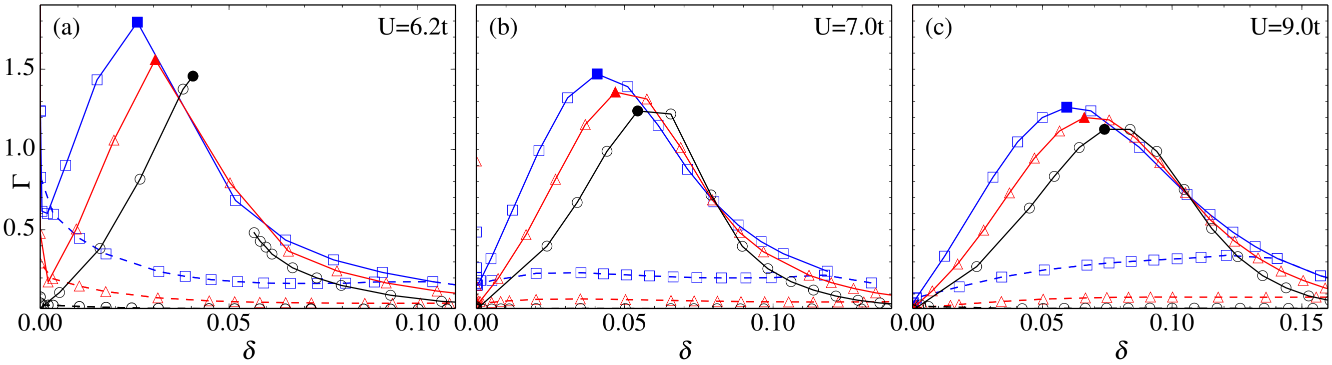

FIG. S3. Scattering rate $\Gamma=-{\rm Im}\Sigma_{(\pi,0)}(\omega\to 0)$ for $U/t=6.2,7,9$ in the normal and superconducting states (full and dashed lines, respectively). The data are shown for temperatures $T/t=1/32$ (blue squares), $1/50$ (red triangles) and $1/100$ (black circles). Interpolation of these data gives rise to the color map in top panels of Fig. 2 of the main text. The maximum of the normal state scattering rate $\Gamma(\delta)|_T$ is marked by a solid symbol and is displayed by solid white diamonds in top panels of Fig. 2 of the main text. Leaving apart the Mott insulator at $\delta=0$, there is a maximum in the normal state $\Gamma(\delta)|_T$ either close to the first-order transition between pseudogap and correlated metal for $T<T_p$ (cf. $U/t=6.2$ and $T/t=1/100$) or in the supercritical region for $T>T_p$ [3, 4]. Upon increasing temperature, the value of $\Gamma(\delta)|_T$ at its maximum increases as does its width in doping. The large scattering rate is sharply depleted upon entering the superconducting state, as already noticed in Refs. [5, 6].

C. PSEUDOGAP TO CORRELATED METAL TRANSITION IN THE NORMAL STATE

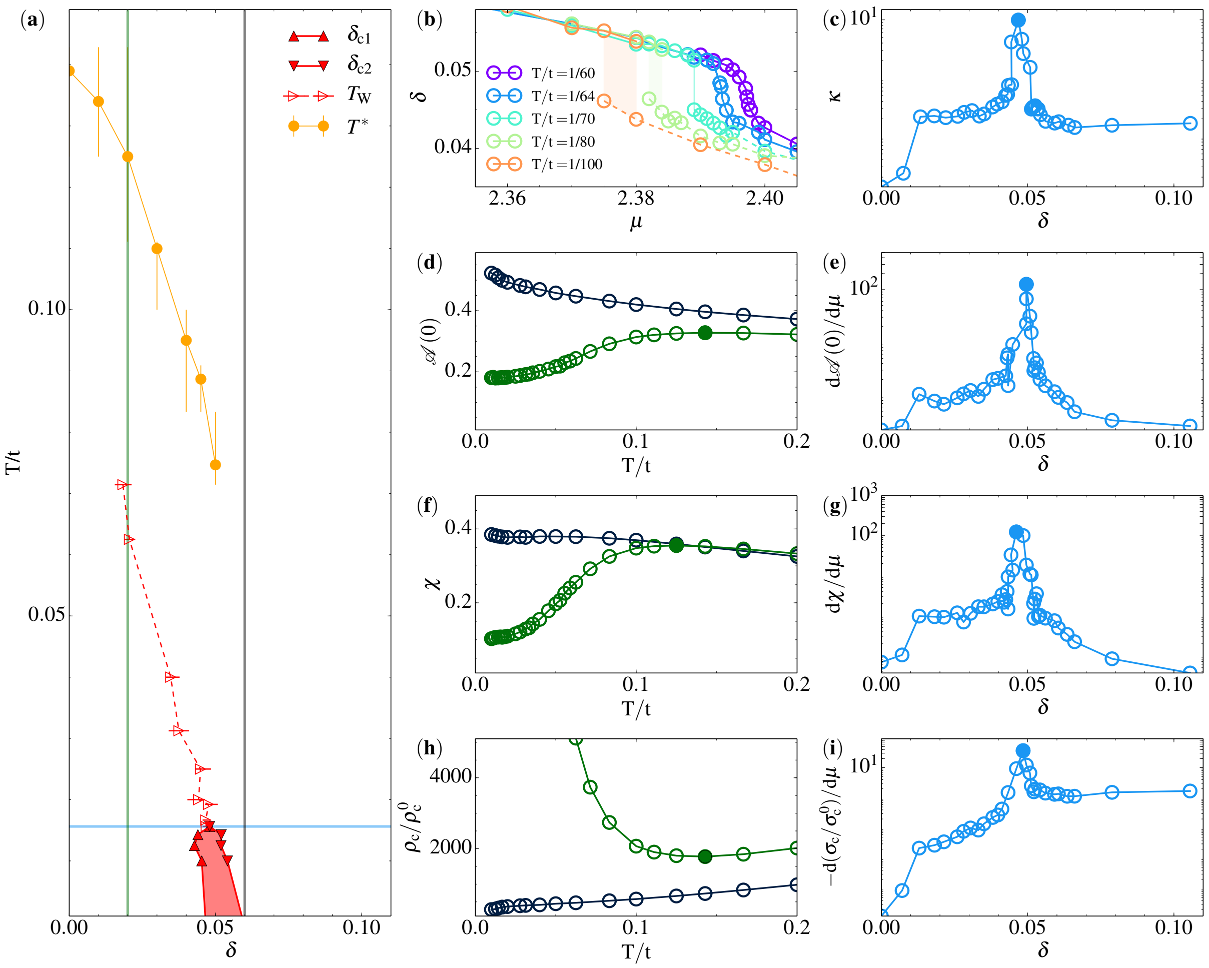

FIG. S4. (a) Temperature versus hole doping phase diagram for $U/t = 6.2$ in the normal state obtained by CDMFT. Data are taken from our previous investigations [1–4]. Horizontal (vertical) shaded lines indicate the values of temperature (doping) of the observables in the other panels. At zero doping, the system is a Mott insulator and is characterised by a plateau in the occupation at $n(\mu) = 1$. At finite doping $\delta = 1 - n$, the coexistence region across a first-order transition between a pseudogap phase and a correlated metal is shown as red shaded area. Its boundaries are obtained by the jumps in the occupation n versus chemical potential μ at constant values of temperature, as shown in panel (b) and discussed in Refs. [3, 4]. Extrapolations to $T = 0$ are a guide for the eye. The pseudogap to correlated metal first-order transition terminates at a critical endpoint $(\delta_p, T_p) \approx (0.045, 1/65)$. Let us first consider paths at constant T [panels (b,c,e,g,i)]. In the supercritical region, $T > T_p$, only one normal-state phase exists and the $n(\mu)$ curves are continuous. The endpoint generates the Widom line T_W (line with red triangles in panel (a)). We estimate T_W by the maxima of the charge compressibility $\kappa = 1/n^2(dn/d\mu)_T$, $\max|_\mu \kappa$ [1]. A semilogarithmic plot of κ versus δ at $T/t = 1/60$ is shown in panel (c), and a filled symbol indicates the position of compressibility maximum. The value of κ at the maximum increases for $T \to T_p$, indicating a divergence of κ at T_p, as investigated in Ref. [1]. The Widom line governs the crossovers of other observables: the local density of states at the Fermi level $\mathcal{A}(\omega = 0)$ [1], the spin susceptibility χ [1], the c-axis DC conductivity σ_c [2], all show inflection points as a function of μ. Their derivative with respect to μ are shown in panels (e,g,i), respectively. Let us now consider scans at constant doping [panels (d,f,h)]. Solely for $\delta < \delta_p$, the temperature dependence of $\mathcal{A}(\omega = 0)$, χ and the c-axis resistivity $\rho_c = 1/\sigma_c$ all show non-monotonic behavior. The position of the minima or maxima in such observables is our estimate for the pseudogap onset. For definiteness, we define T^* (line with orange circles in panel (a)) by the maxima in $\chi(T)$.

D. KINETIC ENERGY IN CDMFT WITHIN HYBRIDIZATION EXPANSION IMPURITY SOLVER

In the hybridization expansion impurity solver, the partition function of the impurity solver is expanded in the hybridization between the impurity and the bath. In single-site DMFT [7], the impurity consists of a site. The kinetic energy per site can be shown [8] to be related with the average expansion order by $E_{\rm kin} = -\langle k\rangle/\beta$, where β is the inverse temperature. Here we generalize this formula for the CDMFT case. We demonstrate that the kinetic energy is the sum of two terms: similarly to the single-site DMFT case, there is a contribution related to the average expansion order term, but there is another term coming from the cluster (plaquette) part. The latter can be computed from the plaquette density matrix (or occupation numbers).

The kinetic energy per site reads

$$E_{kin} = \frac{2}{N}\sum_{i,j}\sum_{r,r'} t_{ij}\left(r-r'\right)\left\langle c_i^\dagger\left(r\right)c_j\left(r'\right)\right\rangle \tag{1}$$

where i,j are indices indicating the position within a cluster, N is the number of sites, and r,r' indicate the position of the cluster. The sum being on all positions and the hopping matrix $t_{ij}\left(r-r'\right)$ being symmetric, there is no need to add the hermitian conjugate. By inserting the definition of the Green function one obtains

$$E_{kin} = \frac{T}{N}\sum_n e^{-i\omega_n 0^-}\sum_{i,j}\sum_{r,r'} t_{ij}\left(r-r'\right)G_{ji}\left(r'-r;i\omega_n\right), \tag{2}$$

and by Fourier transformation on the position of the clusters

$$E_{kin} = \frac{2T}{N}\sum_n e^{-i\omega_n 0^-}\sum_{i,j}\sum_{\widetilde{k}} t_{ij}\left(\widetilde{k}\right)G_{ji}\left(\widetilde{k};i\omega_n\right). \tag{3}$$

We keep a discrete wave vector sum. Using the expression for the inverse of the lattice Green function, the hopping can be rewritten so that

$$E_{kin} = \frac{2T}{N}\sum_n e^{-i\omega_n 0^-}\sum_{i,j}\sum_{\widetilde{k}}\left[i\omega_n+\mu-\Sigma_{ij}\left(i\omega_n\right)-G_{ij}\left(\widetilde{k};i\omega_n\right)^{-1}\right]G_{ji}\left(\widetilde{k};i\omega_n\right) \tag{4}$$

$$= \frac{2T}{N}\sum_n e^{-i\omega_n 0^-}\left[\sum_{i,j}\sum_{\widetilde{k}}\left[(i\omega_n+\mu-\Sigma_{ij}\left(i\omega_n\right))G_{ji}\left(\widetilde{k};i\omega_n\right)\right]-\sum_i\sum_{\widetilde{k}}1\right]. \tag{5}$$

The self-consistency condition is given by

$$G_{ji}^{imp}\left(i\omega_n\right) = \frac{1}{N_{sr}}\sum_{\widetilde{k}} G_{ji}\left(\widetilde{k};i\omega_n\right). \tag{6}$$

where $N_{sr} = N/N_c$, and N_c is the cluster size (here $N_c = 4$). This relation allows one to perform the sum over $\widetilde{k}$ and to write E_{kin} as

$$E_{kin} = \frac{2T}{N_c}\sum_n e^{-i\omega_n 0^-}\left[\sum_{i,j}\left[(i\omega_n+\mu-\Sigma_{ij}\left(i\omega_n\right))G_{ji}^{imp}\left(i\omega_n\right)\right]-\sum_i 1\right], \tag{7}$$

where we used that $\sum_{\widetilde{k}} = N_{sr} = \frac{N}{N_c}$. Inserting the expression for $G_{ij}^{imp}\left(i\omega_n\right)^{-1}$, one obtains

$$E_{kin} = \frac{2T}{N_c}\sum_n e^{-i\omega_n 0^-}\left[\sum_{i,j}\left[(G_{ij}^{imp}\left(i\omega_n\right)^{-1}+t_{ij}^{imp}+\Delta_{ij}\left(i\omega_n\right))G_{ji}^{imp}\left(i\omega_n\right)\right]-\sum_i 1\right] \tag{8}$$

$$= \frac{2T}{N_c}\sum_n e^{-i\omega_n 0^-}\sum_{i,j}\left[(t_{ij}^{imp}+\Delta_{ij}\left(i\omega_n\right))G_{ji}^{imp}\left(i\omega_n\right)\right] \tag{9}$$

$$= \frac{2T}{N_c}\sum_n e^{-i\omega_n 0^-}\sum_{i,j}\left[\Delta_{ij}\left(i\omega_n\right)G_{ji}^{imp}\left(i\omega_n\right)\right]+\frac{2T}{N_c}\sum_n e^{-i\omega_n 0^-}\sum_{i,j}\left[t_{ij}^{imp}G_{ji}^{imp}\left(i\omega_n\right)\right]. \tag{10}$$

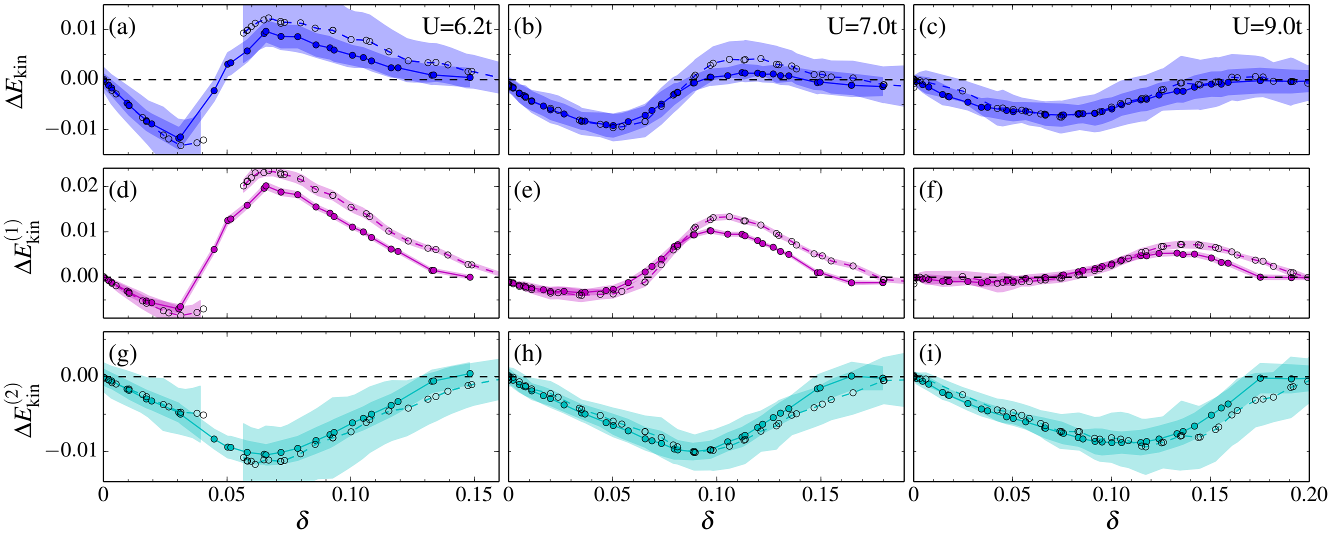

FIG. S5. Different contributions to the difference in kinetic energy between the superconducting and the normal state as a function of doping for $U/t = 6.2, 7, 9$ (left, central and right columns, respectively) and $T/t = 1/50, 1/100$ (full and dashed line, respectively). Top panels: difference in total kinetic energy $\Delta E_{\rm kin}$; Central panels: contribution from terms outside the cluster $\Delta E^{(1)}_{\rm kin}$; Bottom panels: contribution from terms within the cluster $\Delta E^{(2)}_{\rm kin}$. We relate the sign change in $\Delta E_{\rm kin}$ to the sign change in $\Delta E^{(1)}_{\rm kin}$. The various contributions are defined by Eqs. 13 and 14.

Using arguments analogous to those in single-site DMFT, [8] the first term is related to the expansion order [9] while the second contribution is

$$\frac{2T}{N_c}\sum_n e^{-i\omega_n 0^-}\sum_{i,j}\left[t^{imp}_{ij}G^{imp}_{ji}(i\omega_n)\right] = \frac{2T}{N_c}\sum_n e^{-i\omega_n 0^-}\sum_K t^{imp}_K G^{imp}_K(i\omega_n) \tag{11}$$

$$= \frac{1}{N_c}\sum_K t^{imp}_K n^{imp}_K. \tag{12}$$

where n^{imp}_K is the occupation of the cluster momentum K. Finally, the total kinetic energy is given by

$$E_{kin} = -\frac{\langle k\rangle}{N_c\beta} + \frac{1}{N_c}\sum_K t^{imp}_K n^{imp}_K \tag{13}$$

where $\langle k\rangle$ is the average expansion order. This last equation serves to define

$$E_{kin} = E^{(1)}_{kin} + E^{(2)}_{kin}. \tag{14}$$

The bottom panels in Fig. S5 shows that on short distances, namely within the cluster, the kinetic energy $E^{(1)}_{\rm kin}$ is aways lowered upon entering the superconducting state. However, as the middle panels show, the contribution to the kinetic energy gain coming from longer distance, or smaller wave vectors, can change sign.

Finally, Fig. S6 shows that the ratio between the potential energy gain and the kinetic energy gain is $-1/2$ in the underdoped region. That ratio corresponds to the ratio between potential and kinetic energy contained in the exchange energy, namely the term that scales like $J = 4t^2/U$ in the large U limit [10]. It seems that not much energy gain comes from the term in the $t-J$ model describing the hopping of holes. The divergences come from the zero crossings of either the kinetic or the potential energy differences.

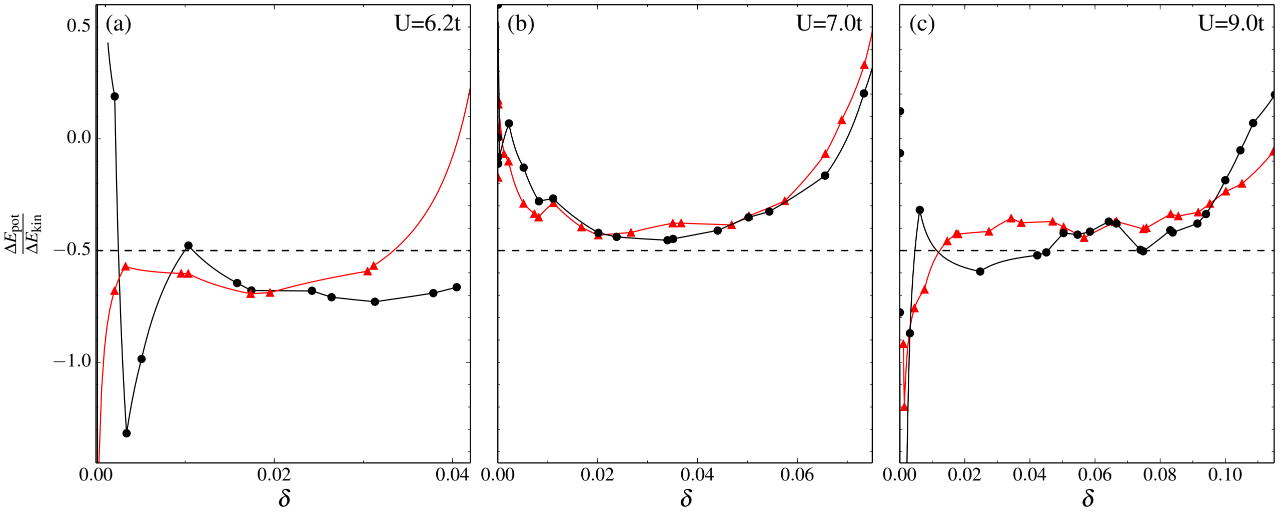

FIG. S6. Ratio between potential energy gain and the kinetic energy gain upon entering the superconducting state in the underdoped region, for $T/t = 1/50$ (red triangles) and $1/100$ (black circles). The horizontal dashed line shows the value $-1/2$ expected from the exchange energy proportional to J.

E. EFFECT OF SECOND-NEIGHBOR HOPPING t'

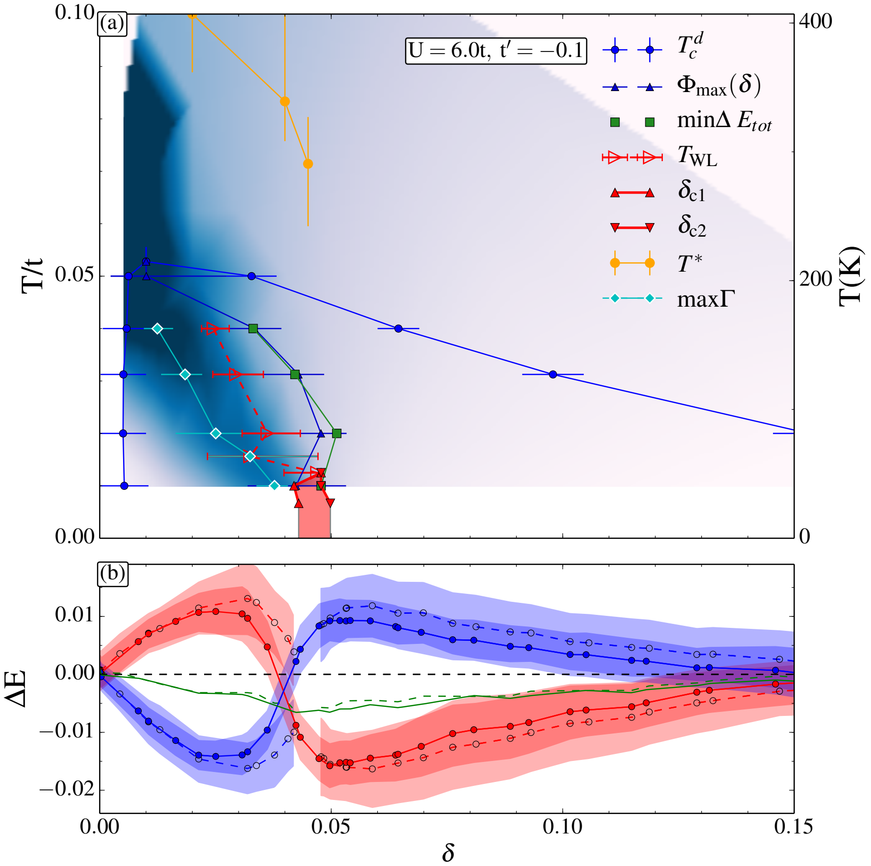

FIG. S7. Same as Fig. 2 of the main text, but for $U = 6.0t$ and $t' = -0.10$. All conclusions remain unchanged with a finite t'.

For $U = 0$, the effect of next-nearest-neighbor hopping t' is to move the van Hove singularity to finite doping. This does have some quantitative effect on the phase diagram at finite U. However for very large U we expect that this is less important. Given that the sign problem is less severe at $t' = 0$ and that values of U can be quite large, the results in the main text are all for $t' = 0$. Nevertheless, we performed calculations for $t' = -0.1$, $U = 6.0$, which is larger than the critical threshold to open a Mott gap at $n = 1$. The results are in Fig. S7. The value of doping where the first-order transition occurs moves to larger doping, as suggested by Fig. 18 of Ref. [11]. But one can verify that our qualitative conclusions concerning the organizing principle of the phase diagram are unchanged. The first order transition in the normal state along with the associated crossovers leave their mark in the superconducting state, even though there is no longer a first-order transition in the superconducting state.

[1] G. Sordi, P. Sémon, K. Haule, and A.-M. S. Tremblay, Sci. Rep. **2**, 547 (2012).
[2] G. Sordi, P. Sémon, K. Haule, and A.-M. S. Tremblay, Phys. Rev. B **87**, 041101 (2013).
[3] G. Sordi, K. Haule, and A.-M. S. Tremblay, Phys. Rev. Lett. **104**, 226402 (2010).
[4] G. Sordi, K. Haule, and A.-M. S. Tremblay, Phys. Rev. B **84**, 075161 (2011).
[5] K. Haule and G. Kotliar, Phys. Rev. B **76**, 104509 (2007).
[6] K. Haule and G. Kotliar, Phys. Rev. B **76**, 092503 (2007).
[7] A. Georges, G. Kotliar, W. Krauth, and M. J. Rozenberg, Rev. Mod. Phys. **68**, 13 (1996).
[8] K. Haule, Phys. Rev. B **75**, 155113 (2007).
[9] The relation to the expansion order is most easily seen in the action formalism.
[10] P. Fazekas, *Lecture Notes on Electron Correlation and Magnetism* ((World Scientific, Singapore), 1999).
[11] A. Liebsch and N.-H. Tong, Phys. Rev. B **80**, 165126 (2009).
